# *Phe362Tyr* in AChE: A Major Factor Responsible for Azamethiphos Resistance in *Lepeophtheirus salmonis* in Norway

**DOI:** 10.1371/journal.pone.0149264

**Published:** 2016-02-16

**Authors:** Kiranpreet Kaur, Peder Andreas Jansen, Vidar Teis Aspehaug, Tor Einar Horsberg

**Affiliations:** 1 NMBU School of Veterinary Science, Sea Lice Research Centre, PO Box 8146 Dep., NO-0033, Oslo, Norway; 2 Norwegian Veterinary Institute, Pb 750, N-0106, Oslo, Norway; 3 PatoGen Analyse AS, Postboks 1527, 6025, Ålesund, Norway; Institute of Zoology, Chinese Academy of Sciences, CHINA

## Abstract

Organophosphates (OP) are one of the major treatments used against the salmon louse (*Lepeophtherius salmonis*) in Norwegian salmonid aquaculture. The use of OP since the late 1970s has resulted in widespread resistant parasites. Recently, we reported a single mutation (*Phe362Tyr*) in acetylcholinesterase (AChE) as the major mechanism behind resistance in salmon louse towards OP. The present study was carried out to validate this mechanism at the field level. A total of 6658 salmon louse samples were enrolled from 56 different fish farms across the Norwegian coast, from Vest Agder in the south to Finnmark in the north. All the samples were genotyped using a TaqMan probe assay for the *Phe362Tyr* mutation. A strong association was observed between areas with frequent use of the OP (azamethiphos) and the *Phe362Tyr* mutation. This was confirmed at 15 sites where results from independently conducted bioassays and genotyping of parasites correlated well. Furthermore, genotyping of surviving and moribund parasites from six bioassay experiments demonstrated a highly significant negative correlation between the frequency of resistance alleles and the probability of dying when exposed to azamethiphos in a bioassay. Based on these observations, we could strongly conclude that the *Phe362Tyr* mutation is a major factor responsible for OP resistance in salmon louse on Norwegian fish farms.

## Introduction

Norwegian government regulations require a reduction of the salmon louse (*Lepeophtheirus salmonis*) burden if the abundance exceeds 0.5 adult female parasites per fish on salmon farms. Due to the limited number of chemotherapeutants available, and the shortage of other control options, antiparasitic treatments that have been used to control *L*.*salmonis*, and against which the parasite has developed resistance, are still in use. The lack of effective alternatives has—and is continues to- a growing resistance problem. Even though some surveillance programs have been conducted to educate fish farmers, fish health services and government bodies about the growing problem [1, 2], a lot still needs to be done.

In order to control the resistance problem in the salmon louse, it is important to diagnose it first. If the resistance is detected at an early stage, better management strategies could be used to control its spread. Unfortunately, the attempts so far have failed to attain this goal.

Organophosphates, inhibitors of the enzyme acetylcholine esterase (AChE) which is responsible for terminating the effect of nerve impulses in cholinergic synapses, have been used for treatment against the marine ectoparasitic copepod salmon louse (*L*. *salmonis*) in Norwegian salmonid aquaculture since the late 1970s. The first agent used was metrifonate (Neguvon^™^), followed by dichlorvos (Nuvan^™^) in 1986 [3] and azamethiphos (Salmosan^™^) in 1994 [4]. In the early 1990s, the first cases of reduced efficacy of organophosphate treatments were noted in Mid-Norway [5]. This reduced sensitivity led to the termination of azamethiphos use in 1999. Even though the resistance towards azamethiphos was widespread, the cause of resistance was unknown. Azamethiphos was re-introduced as a treatment agent against the salmon louse in 2008 [6]. New reports of reduced efficacy of azamethiphos treatments came from the field in 2009. In 2013 and 2014, a surveillance program, using bioassays to test for resistance, revealed a widespread distribution of azamethiphos resistance on Norwegian fish farms [1,2].

Recently, two genes coding for AChE in *L*. *salmonis* were identified and characterized [7]. The two AChEs in *L*. *salmonis* are highly similar to each other (84% similarity at the protein level), an observation quite unique to *L*. *salmonis* and previously has not been observed in other arthropods. The two AChEs were denoted as *ace1a* and *ace1b*. *Ace1a* was predominantly expressed in the different developmental stages of salmon louse compared to *ace1b* and was active in the cephalothorax, indicating that *ace1a* plays a major role in synaptic transmission. In addition, the screening of both *ace1a* and *ace1b* in resistant strains (5 strains) and sensitive strains (2 strains) led to the identification of a single missense mutation only in *ace1a*, leading to a change from phenylalanine to tyrosine at codon 362 [8]. The significantly higher frequency of *362Tyr* in *L*. *salmonis* samples resistant to azamethiphos indicated a clear association of *Phe362Tyr* with reduced sensitivity towards azamethiphos. Furthermore, 3D modelling, small scale treatment trials for genetic characterization with azamethiphos and enzymatic inhibition assays provided a strong argument in favor of *Phe362Tyr* mutation being the culprit behind azamethiphos resistance in *L*. *salmonis* [8].

However, the above conclusions were based on observations from experiments conducted on laboratory cultivated strains. The aim of this study was to explore the bigger picture: the scenario on fish farms across the entire Norwegian coast. What role does *Phe362Tyr* play in the *L*. *salmonis* resistance towards OP throughout the country? Is it a major factor responsible for OP resistance in Norway? Or is there a more complex story behind it? These intriguing questions provided the basis of the present study where we attempted to find answers to the above questions.

## Materials and Methods

This epidemiological study of genotypes associated with sensitivity towards azamethiphos in salmon louse was based on salmon louse samples submitted to the laboratory by salmon-producing companies, and bioassays conducted under the national resistance monitoring program by various fish health services along the Norwegian coast [2].

### Samples

A total of 6658 salmon louse samples (preadult and adult stages) were submitted to the laboratory from 56 different fish farms over the period of 2012–2014 (one farm was sampled twice). Most samples (4548) were submitted from 41 random farms where there was no information about the treatment history or sensitivity status of the parasites towards azamethiphos. A second set of samples (1696) was submitted from 15 sites included in the national monitoring program for salmon louse resistance [1], however not at the same time as the survey. Thus, for these samples, bioassay results based on a total of 1336 samples were also available. These were two-dose bioassays, which had been conducted independently by local fish health services. The third set of samples (414) included parasites originating from sites incorporated in the resistance monitoring program (6 sites) and were the parasites used in the bioassays in this survey. After exposure to 0, 0.4 or 2 ppb (μg/L) azamethiphos for 24 hours [9], samples were separated into alive and moribund parasites. For these samples, both the genotyping results and the individual phenotypic response to azamethiphos exposure (alive or moribund) were available.

The salmon lice were collected as per the instructions provided by PatoGen Analyse AS and stored in 70% ethanol or RNAlater at -20°C until analysis. All samples were genotyped by PatoGen Analyse AS.

### Genotyping

A standard TaqMan assay was developed by PatoGen Analyse AS for the rapid and high throughput screening of the mutation (*Phe362Tyr*) identified in *ace1a* [8].

On basis of this assay, each parasite could be classified as homozygote wild type (SS; *Phe362*/*Phe362*), heterozygote (RS; *362Tyr/Phe362*) or homozygote mutated (RR; *362Tyr*/*362Tyr*).

### Statistical analyses and modeling

The statistical analyses comprised of three different sets of data with accompanying analyses, which we termed the spatial, bioassay and individual louse analysis, respectively.

#### Spatial analysis

The dataset accompanying the spatial analysis consisted of the 6658 genotyped samples of salmon louse originating from 56 farms along the coast (see S1 Table). The questions that were addressed in the analysis of these data were 1) whether there was a predictable spatial pattern in the distribution of mutated *362Tyr* genotypes? and 2) whether this distribution was associated with farm treatments or local treatment density?

**Fig 1:**
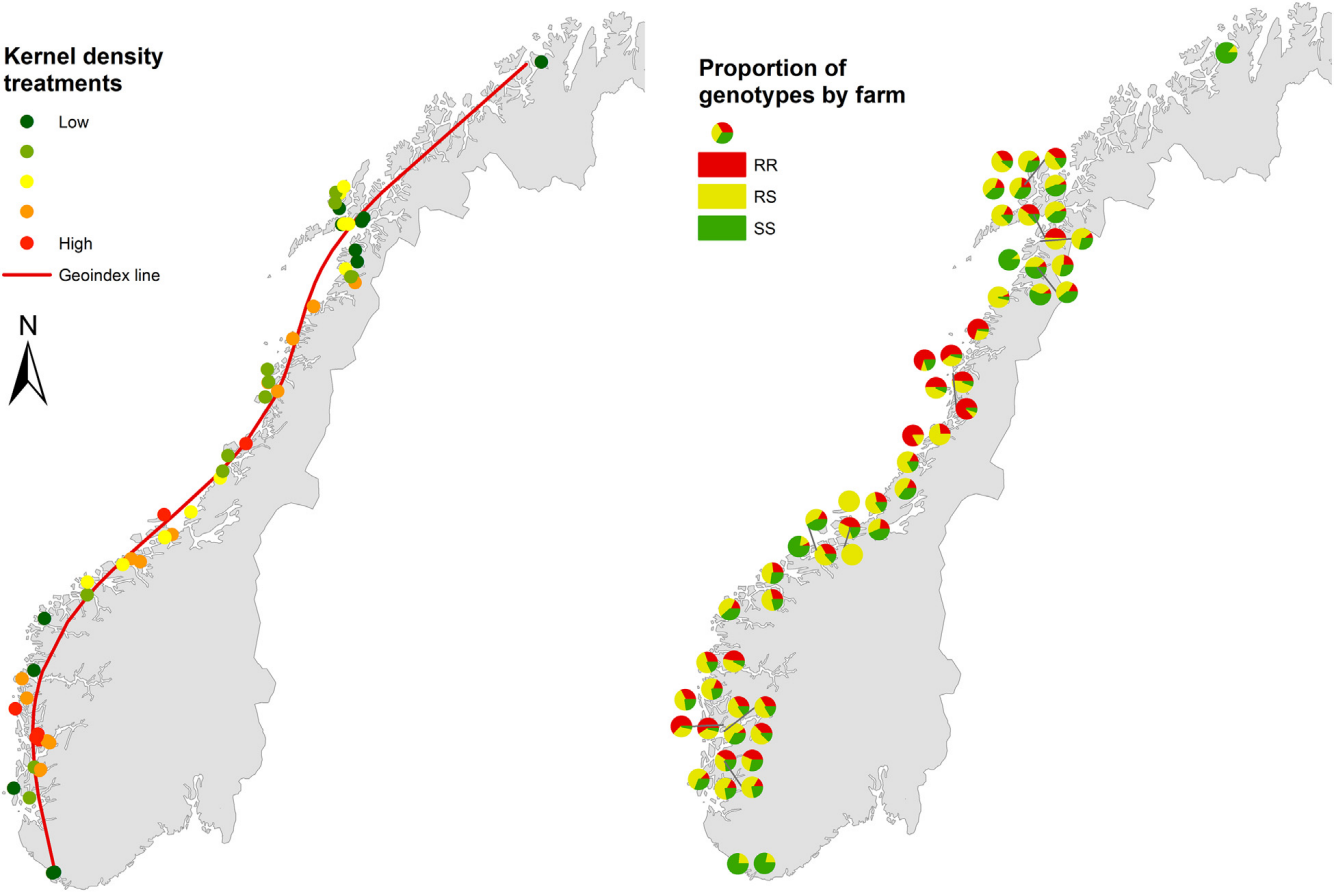
Relative quantities of the kernel density of treatments along with the geoindex line. Maps showing relative quantities of the kernel density of treatments along with the geoindex line (left panel), and the proportion of the SS, RS and RR genotypes (right panel, note that locations of farms are displaced in areas with clusters of farms to avoid overlapping symbols).

To test whether there was a predictable spatial pattern in the distribution of mutated genotypes, we first assigned a geographical index (geoindex) to each sampled farm. The geoindex accounts for both longitude and latitude using the method presented in Kristoffersen et al. [10]. This geoindex orders the farms in a south to north gradient and additionally accounts for their east to west gradient. The geoindex was constructed using a local polynomial regression [11], where the dependent variable comprised of farm latitudes and the independent variable of farm longitudes. Each farm was then projected onto the regression line and attributed a geoindex according to the expression given in Kristoffersen et al. [10]. The geoindex regression line is shown in Fig 1.

To test if the distribution of mutated *362Tyr* genotypes was associated with farm treatments using azamethiphos, or local densities of such treatments, reported treatment data were compiled. Reporting of bath treatments to the Norwegian Food Safety Authority according to categories of substances is mandatory, including azamethiphos, deltamethrin, and cypermethrin, or as “other”. Here we summarize the number of reports categorized as azamethiphos for every reporting salmon farm for each of the years 2012–2014.

It is mandatory to treat all pens on the farm when using a bath treatment, [12]. This takes time and treatment reports from given farms are often repeated over consecutive weeks. To summarize the accumulated number of bath treatments per year, we regarded sequences of treatments over consecutive weeks to count as one treatment. The number of farms reporting the use of azamethiphos, the total number of treatment reports and the accumulated number of treatments are summarized in Table 1. The number of treatments for the farms sampled for salmon louse for the given sampling year was extracted from the summary number of treatments (Table 1) and given in S1 Table.

**Table 1:** The number of farms reporting the use of azamethiphos, the total number of treatment reports and the accumulated number of treatments, for the years 2012–2014.

Year	Farms	Total number of reports	Number of treatments
2012	244	601	422
2013	204	486	311
2014	290	568	479

The numbers of treatments per farm per year were used to calculate local densities of treatment. For each year between 2012–2014, all treatment reporting farms were projected onto a map in ArcView (ESRI, Redlands, CA, USA) using the projection WGS84n33. Spatial kernel densities of treatment were then calculated for the number of treatments per farm, using the Kernel Density tool in the ArcView extension Spatial Analyst (ESRI, Redlands, CA, USA), a grid size of one by one km and search radius of 25 km. The kernel density quantities for the farms sampled for salmon louse were then extracted by the “Extract values to points” tool in Spatial Analyst. Kernel densities of treatment for sampled farms in the given year of sampling are given in S1 Table.

For the statistical analysis of the spatial data we used the proportion of lice within unique farm samples in given years that harbored a mutation for azamethiphos resistance, *i*.*e*. p = (RR+RS)/(RR+RS+SS), as the dependent variable. Since proportions are not normally distributed, the dependent variable was transformed by taking the arcsin of the square root of the proportion [13]. For the analysis, we used general additive models (GAM) of the form:

$$arcsin\sqrt{p}=\beta_{0}+\;\beta_{1}fX_{1}+\;\beta_{n}X_{n}$$

where $arcsin\sqrt{p}$ is the dependent variable, *β*_0_ is the intercept, *f*_1_ denotes a smooth function of the spatial variable *X*_1_ with coefficient *β*_1_, and *X*_*n*_ represents any other co-variate entered in the model with coefficients *β*_n_. GAM was used to allow for non-linear effects of the spatial variables. Since the variable expressing the number of treatments per farm was highly skewed, it was entered as a factorial variable with three classes representing 0, 1 or >1 treatments. Also the kernel density of treatments was skewed, so it was entered as a square root transformed variable in the analyses, yielding a reasonably symmetric distribution.

The analysis was done using the “mgcv” library in R [14]. First, we tested the variables in univariate models. Then we added variables in a forward selection procedure, while also checking for correlation between variables.

### Bioassay analysis

The dataset accompanying the bioassay analysis consisted of 1336 salmon lice from 15 different salmon farms (see S1 Table), which were tested in bioassays with the outcome moribund or alive at concentrations of 0, 0.4 or 2 ppb azamethiphos, respectively. The question that was addressed in the analysis was whether the farm proportions of lice with a susceptible genotype (SS), and the concentration of azamethiphos in the bioassay tests, contributed to predict the test outcome. The control experiments with concentration = 0 gave less than 4% mortality, and hence these were omitted from further assessments.

To assess whether farm level susceptibility and azamethiphos concentration predicted the outcome of bioassay tests for individual salmon louse, a multivariable mixed effects logistic model was developed. The model had the form:

$$\text{logit}P\left(Yi=1|X\right)=\;\beta_{0}+\beta_{1}\sqrt{p.SS}+\beta_{2}\text{conc}.\;+\;\omega \;\text{Farm}$$

where the dependent variable expresses the binomial outcome (moribund or alive) on a logit scale for individual parasites, *β*_0_ is the intercept, $\sqrt{p.SS}$ denotes the square root of the proportion of SS genotypes on farms with coefficient *β*_1_, *conc*. is a two level factor (0.4 and 2 ppb) accounting for the azamethiphos concentration with coefficient *β*_2_ and *Farm* is a random effect of farm with coefficient *⍵*. The intra-class correlation coefficient (ICC) was calculated according to Dohoo et al. [13] as:

$$\sigma^{2}{}_{\text{Farm}}/(\pi^{2}/3\;+\;\sigma^{2}{}_{\text{Farm}})$$

where σ^2^_Farm_ represents the variance between farms adjusted for the assumed variance between lice within farm (π^2^/3).

#### Individual louse analysis

To further assess the phenotype of different genotypes with respect to azamethiphos susceptibility, bioassays were conducted with lice sampled from six different farms where the outcome (moribund or alive) was classified for individual parasites. All the individual salmon lice were subjected to 24-hour bioassays at either 0.4 or 2 ppb azamethiphos concentration, along with a control group (0 ppb). After classification, individual parasites (n = 441, see S1 Table) were collected and later genotyped for the *Phe362Tyr* mutation. The question that was addressed in the analysis was how do individual genotypes, along with different concentrations of azamethiphos, predict the outcome (moribund or alive) of bioassay tests.

The bioassay tests with individual genotyping of lice post exposure were first analyzed in a logistic model similar to that of the bioassay analysis, with farm (n = 6) as random effect. There was, however, no significant variance that was explained at the farm level in this model. Hence, these data were analyzed with logistic regression with only fixed effects, in a model of the form:

$$\text{logit}P\left(Yi=1|X\right)=\;\beta_{0}+\beta_{1}Genotype\;+\beta_{2}\mathrm{conc}.$$

where Genotype is an individual louse factor of three levels (SS, RS and RR) and conc. is a three level factor accounting for azamethiphos concentration (0, 0.4 and 2 ppb).

### Ethical statement

The study only involved the salmon louse and no fish were being used. All the parasites were collected from the fish farms (locations mentioned in the Materials and Methods) with the farmers' consent.

After farmers consent, no special permissions were required for the sample collection. The study did not involve endangered or protected species.

## Results

### Spatial analysis

The 6658 salmon louse samples in this study were enrolled from fish farms distributed throughout the Norwegian coast, from Vest Agder in the south to Finnmark in the north (Fig 1). These areas represent a large part of the salmon-producing coast in Norway. All the 6658 samples were genotyped for *Phe362Tyr* mutation using TaqMan probe analyses. Screening of the samples revealed that *Phe362Tyr* was widespread throughout the Norwegian coast. The proportion of different genotypes (SS, RS and RR) for the *Phe362Tyr* mutation in different areas is shown in Fig 1, right panel. For the whole Norwegian coast per se, the proportion of salmon louse without *Phe362Tyr* mutation (i.e. the SS genotype) was significantly lower (23.1%) than the salmon lice with *Phe362Tyr* mutation (76.9%, of which 42.2% were RS and 34.7% RR, respectively). The frequencies of the different genotypes per county are given in Table 2.

**Table 2:** Percentage distribution of the *Phe362Tyr* mutation (RS and RR genotypes) versus the wild type (SS genotype) in the samples collected from fish farms along the Norwegian coast.

County	SS	RS	RR	Total number
Finnmark	86.4%	13.6%	0.0%	118
Nordland	20.9%	40.7%	38.4%	2320
Nord Trøndelag	20.5%	48.0%	31.5%	337
Sør Trøndelag	23.2%	44.2%	32.6%	362
Møre & Romsdal	27.9%	45.8%	26.3%	567
Sogn & Fjordane	21.4%	48.9%	29.7%	276
Hordaland	13.5%	43.3%	43.2%	2140
Rogaland	25.4%	59.2%	15.4%	240
Vest Agder	77.5%	20.8%	1.7%	298

In the spatial analysis, the factor year was not significantly associated to the proportion of mutated *362Tyr* genotypes, and was therefore excluded from further models.

The variables expressing farm treatments and local density of treatment (Kernel density) were both significantly associated with the proportion of mutated *362Tyr* genotypes in uni-variable models (Pearson r = 0.53). However, when both variables were entered as linear terms into the statistical model, the factorial farm treatments was no longer significant (F = 0.71, p = 0.5) and was therefore excluded from further models.

**Fig 2:**
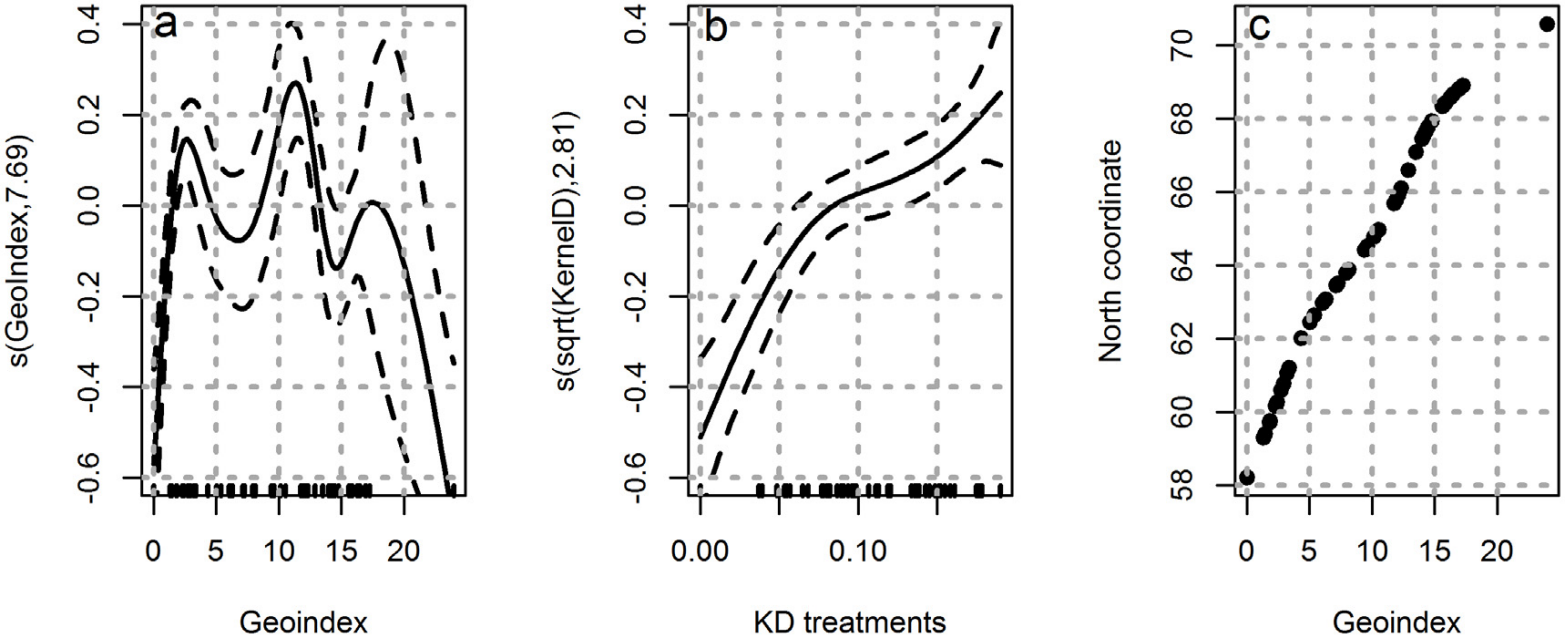
Effects of the geoindex or the kernel density of treatments (KD) on the proportion of mutated genotypes. GAM-plots showing the non-linear relative effects of the geoindex (left panel) or kernel density of treatments (KD) on the proportion of mutated genotypes (RR or RS) in farm sampled salmon lice. The two plots represent univariate fitting of the proportion model. The relationship between farm geoindexes and their north coordinate is also shown (right panel).

The geoindex variable entered as a non-linear smooth term was strongly associated with the proportion of *Phe362Tyr* mutation, accounting for 56% (adj. R^2^ = 0.561) of the variance in the dependent variable (p < 0.001, AIC = -5.48). As shown in Fig 2a, the proportion of *Phe362Tyr* mutation was lowest in the areas with the lowest and the highest geoindex values. These areas correspond to Vest Agder (lowest geoindex value) and Finnmark (highest geoindex value) counties, respectively.

When the kernel density of azamethiphos treatments was entered as a non-linear smooth term in the statistical model, it was also strongly associated with the proportion of *Phe362Tyr* mutation, accounting for 44% (adj. R^2^ = 0.444) of the variance in the dependent variable (p < 0.001, AIC = 3.69). The square root of the kernel density of treatments yielded a close to linear relationship with the dependent variable (Fig 2b).

The fish farms in the areas with the geoindex ~12 that correspond to latitude of about 65.0 decimal degrees north had the highest proportion of *Phe362Tyr* mutation (Fig 2a and 2c). These areas correspond to Nord Trøndelag county. Predictions from the univariate model yielded min and max expected proportions of mutated genotypes amounting to 0.14 and 0.95, respectively, along the geoindex line (given by min and max of: sin (model prediction)^2^).

When both the geoindex smooth term and a linear kernel density of treatment term were entered together in the GAM model, the treatment variable became slightly non-significant (p = 0.055, Table 3). Predictions for the dependent variable using the univariate geoindex model, however, correlated closely with the kernel density of treatments (correlation coefficient = 0.47), indicating that the geoindex effect is closely associated with local treatment densities.

**Table 3:** Statistics for a model entering a non-linear function of geoindex and kernel density of treatments (KD) as predictors for the dependent variable expressing the proportion of mutated genotypes in salmon louse samples from farms (adj. R^2^ = .573, AIC (-6.51)). Coeff refers to regression coefficient and SEM to standard error of mean.

	Coefficient	SEM	P value
Intercept	0.92	0.087	< 0.001
KD	1.51	0.77	0.055
Smooth term	edf	Ref.df.	
GeoIndex	7.48	8.38	0.003

### Bioassay analysis

Both the treated groups (0.4 ppb and 2 ppb azamethiphos) had higher mortality rates than the control (0 ppb azamethiphos) group in the bioassay tests (Table 4). The mortality rate was on average 28% in the 0.4 ppb groups, and 43% in the 2 ppb groups. At both 0.4 ppb and 2 ppb, all fully sensitive parasites (SS) are expected to be immobilized [9].

**Table 4:** A summary of outcomes of salmon louse viability in bioassay tests at different concentrations of azamethiphos. The average % immobilized and alive parasites in the 15 assays, and their standard deviation, are given. The total number of parasites are also given.

Concentrations ppb (μg/L)	Immobilized Avg. % (SD)	Alive Avg. % (SD)	Number (n)
0.0	4 (5)	96 (5)	430
0.4	28 (27)	72 (27)	460
2.0	43 (29)	57 (29)	446

Both the proportion of susceptible lice on farms and the two levels of azamethiphos concentration were significantly associated with the outcome of the bioassay tests (Table 5).

**Table 5:** Final mixed logistic model for lice-level mortality in bioassay tests (Coeff refers to regression coefficient and SEM to standard error of mean). AIC in the full model was 17889.

Parameters	Level	Coeff	SEM	p
Intercept (*β*_0_)		-3.2676	0.6015	< 0.001
Susceptibility (prop.) (*β*_2_)		4.8554	1.2496	< 0.001
Concentration (*β*_3_) (ppb)	0.4	0		
Concentration (*β*_3_) (ppb)	2	0.8074	0.1617	< 0.001
Random effects (*ѡ*) Farm		σ^2^_Farm_		
Random effects (*ѡ*) Farm		0.7968	0.2305	

The intra-class correlation coefficient (ICC) was 0.19 (95% CI: 0.09–0.28), indicating that approximately 19% of the variance was at the farm level.

**Fig 3:**
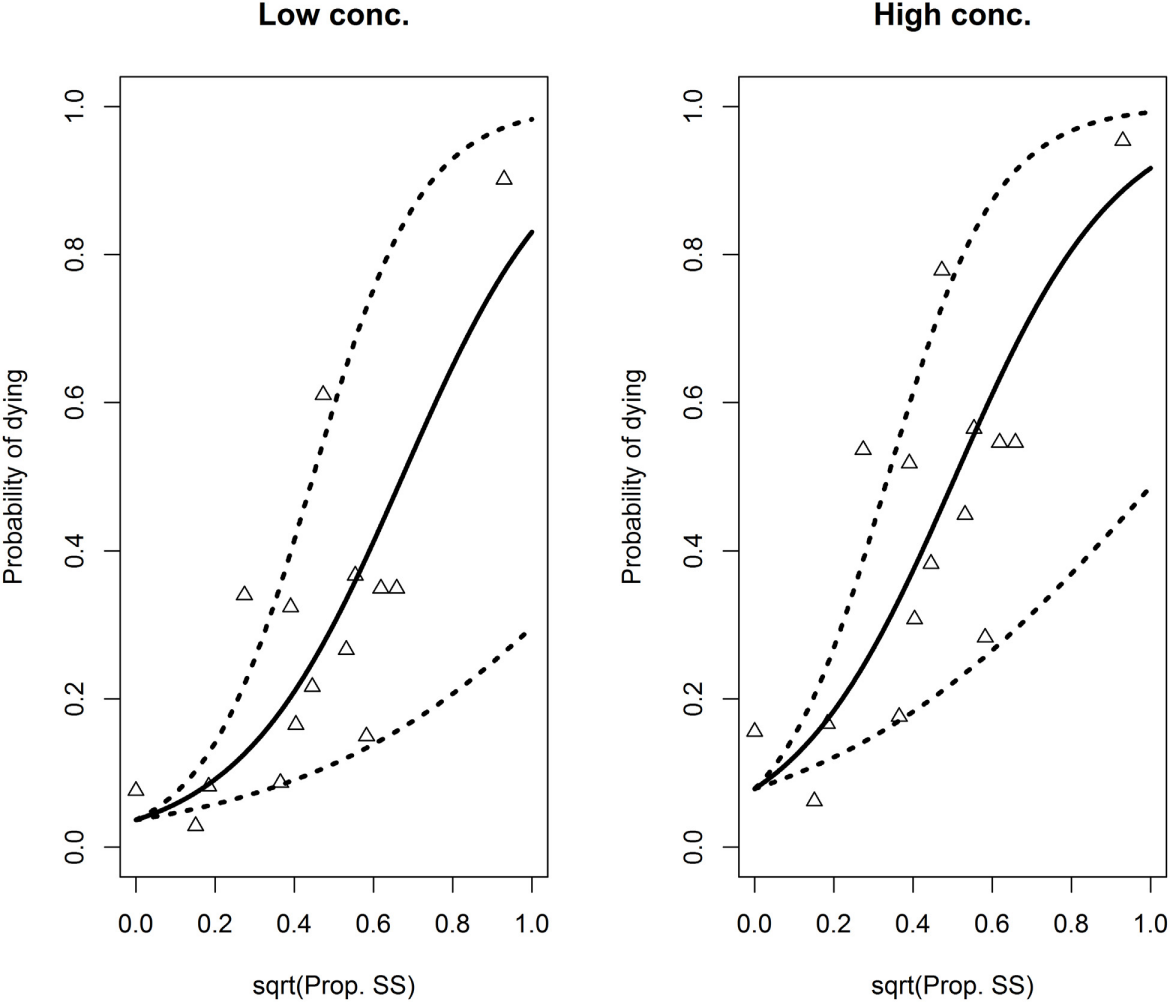
Relationship between the susceptible genotype (SS) and the death of parasite under azamethiphos treatment. The probability of salmon louse dying as a function of the proportion of susceptible salmon louse on farms (square root transformed), when exposed to low (0.4 ppb, left panel) or high (2.0 ppb, right panel) concentrations of azamethiphos, respectively. The lines represent model predictions using the fixed effects (the dotted lines account for the 95% CI for the susceptibility-coefficient). The points represent farm-specific model predictions accounting for the random effects.

The predicted probability of salmon louse dying in the bioassay tests increased from close to zero to ~ 0.9 with increasing proportions of SS-genotypes in the farm populations of salmon louse (Fig 3). The main difference between the low and the high concentration was a steeper increase in the estimated probabilities of dying with increased proportion of SS genotypes at the high concentration. Fig 3 also shows the farm specific predictions for the probability of lice dying when accounting for the farm random effects, which deviates moderately from that of the fixed effect model.

### Individual louse analysis

**Fig 4:**
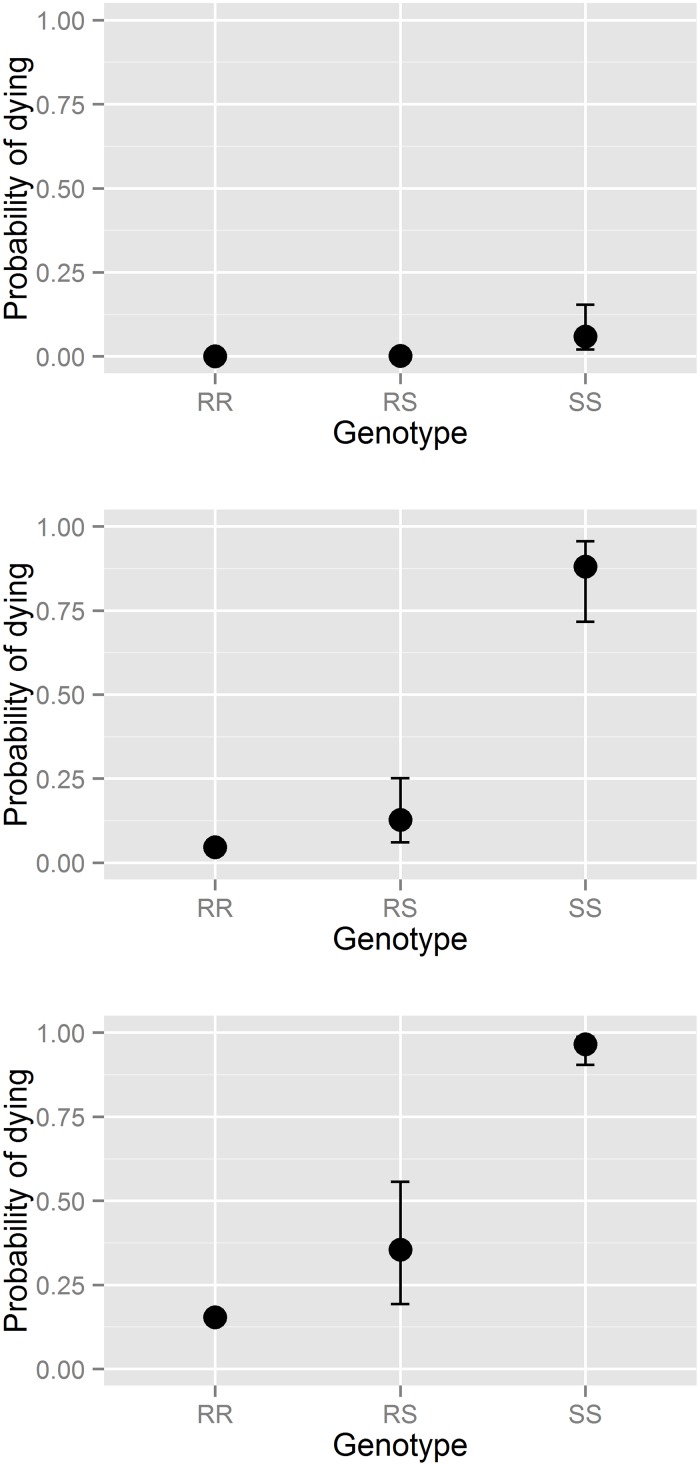
Relationship between different genotypes and the probability of dying under azamethiphos treatment. The estimated probability of salmon louse of various genotypes dying in bioassay tests when exposed to 0 (control) ppb (upper panel), 0.4 ppb (mid panel) and 2.0 ppb (lower panel) azamethiphos, respectively. The error-bars account for the 95% CI for the genotype coefficient.

The probability of individual salmon louse dying was significantly associated to both genotype and azamethiphos concentration in the bioassay-tests conducted for the individual louse outcome (Table 6). The statistical model predicted that lice without the *Phe362Tyr* mutation (SS) would practically only survive in the control experiments (0 ppb azamethiphos), whereas the RR genotype would in general survive all exposures (0, 0.4 and 2 ppb azamethiphos). The RS genotype was intermediately susceptible, with low estimated mortality in the low concentration experiments, but increased mortality in the high concentration experiments (Fig 4).

**Table 6:** General linear model (GLM) for the binomial outcome of salmon louse exposure to azamethiphos in bioassay tests tests (Coeff refers to regression coefficient and SEM to standard error of mean).

Parameters	Level	Coeff	SEM	p
Intercept (*β*_0_)		-7.8025	0.9026	< 0.001
Genotype	RR	0		
Genotype	RS	1.1044	0.4223	0.008
Genotype	SS	5.0248	0.5472	< 0.001
Concentration	0	0		
Concentration	0.4	4.7769	0.8333	< 0.001
Concentration	2.0	6.0988	0.8602	< 0.001

## Discussion

Recently, we identified the mechanism behind resistance in *L*. *salmonis* towards azamethiphos. A single mutation (*Phe362Tyr*) in acetylcholinesterase gene was responsible for resistance in salmon louse towards azamethiphos [8].

The present study was conducted to validate the above findings at the field level. In order to achieve the goal, a rapid, high throughput diagnostic assay was developed by PatoGen Analyse AS, based on the findings of Kaur et al. [8]. Fish farms covering almost the complete Norwegian coast were enrolled in the study (Fig 1). A large cohort of salmon louse (6658) from these farms was collected for *Phe362Tyr* screening using this TaqMan assay.

The farms enrolled in the study had a wide range of resistance levels, from almost completely sensitive areas (Finnmark and Vest Agder) to areas with grave resistance problems (Nordland, Nord Trøndelag, Hordaland), in order to depict association of *Phe362Tyr* with different grades of resistance towards azamethiphos.

The TaqMan probe analyses revealed a wide distribution of *Phe362Tyr* across the Norwegian coast. The frequency of resistant genotypes (RS and RR) was higher (42% RS, 35% RR) than the proportion of samples that did not harbor the mutation (23% SS). Only 5 of the 56 farms enrolled had an SS frequency of 70% or more, whereas 37 had an SS frequency of less than 30%. Four farms had an RR frequency of 70% or more. The RS genotype, with one mutant allele, was the most predominant genotype across the coast, being detected in samples from all 56 farms enrolled. This clearly demonstrates that the *Phe362Tyr* mutation is extremely widespread in Norway, although the spatial distribution varies (Fig 1).

The probability of finding the *Phe362Tyr* mutation was the highest in the areas with geoindex 12 that corresponds to latitude of about 65.0 decimal degrees north, i.e. Nord Trøndelag (Fig 2). Interestingly, these observations are very much similar to the surveillance programs that were conducted to determine the resistance of salmon louse towards various chemotherapeutants in Norway [2]. In this surveillance program, the authors have concluded that resistance in salmon louse towards azamethiphos is widely spread across the coast with just Finnmark and Vest Agder as exceptions with high sensitivity towards the chemical. Taken together, the results from the present study and the surveillance program, we could strongly suggest that *Phe362Tyr* mutation is a major factor responsible for the resistance in salmon louse towards azamethiphos along the Norwegian coast.

The predominance of RS genotype could be explained by the hypothesis suggested by Shi and coworkers [15], which states that there is a fitness cost associated with mutations in AChE, conferring resistance towards OP. But despite the fitness cost, the mutant allele might still prevail in the population without selection (treatment) pressure. However, it depends on the alteration in the protein caused by the mutant allele. The point mutations cause a low level of alteration in the protein, which is the main driving force responsible for the maintenance of resistant alleles in natural populations [15]. The fact that most of the mutations reported in AChE are point mutations supports the above suggestions [16]. The samples for the present study were enrolled at different time points (from 2012 to 2014). The areas with RS as the predominant genotype could be the ones that were not under treatment pressure at the time of enrollment and the ones with higher RR genotype would be under treatment pressure and the population is already selected for the mutant allele. Alternatively, the areas with higher RR proportion would have already reached a stage where the mutant allele is almost fixed in the population.

Our previous study has also provided a good evidence to support this hypothesis [8], where we compared the frequencies of *Phe362Tyr* in the salmon louse population before and after azamethiphos treatment. The frequencies of samples with *Phe362Tyr* increased from 56% to 100% in the population without and under azamethiphos treatment, respectively. Besides, we found the heterozygous status of *Phe362Tyr* in samples that were collected from 1998. This again ascertained the presence of mutant allele in the salmon louse population, without selection pressure, for eight (2000–2007) years. Based on these observations, we could possibly surmise that RR frequency would predominate when these populations are exposed to frequent subsequent azamethiphos treatments, which eventually would lead to a significant reduction or almost complete loss of the SS genotype.

The kernel density of treatments was found to be a strong predictor of the spatial distribution of *Phe362Tyr* mutation in salmon farms sampled for salmon louse along the Norwegian coast (Fig 2). A near to linear relationship was observed between the kernel density of treatments assigned to sampled farms and the proportion of lice with mutation (*Phe362Tyr*). Nevertheless, when the geoindex variable was entered as a predictor of farm proportions of mutated genotypes (RS +RR), together with the kernel density of treatments, the latter variable became slightly non-significant. This, we suspect, could be due to the co-linearity between the two spatial variables. We do therefore believe that the association between local intensities of treatment and the distribution of mutated genotypes that is seen in the present data supports the hypothesis that treatments and the development of resistance in the salmon louse are linked. The geoindex-effect may be confounded by the same mechanisms as the local treatment effect. The geoindex effect is, nevertheless, informative with regard to illustrating how the occurrences of mutated genotypes vary along the coast.

A subset of samples (1336) was subjected to bioassays to correlate the genotype (*Phe362Tyr*) with the phenotype (alive /moribund) at the population level in 15 farms. A near to linear correlation between the probability of dying and the SS genotype further strengthened the role of the *Phe362Tyr* mutation in the survival of parasite under azamethiphos treatment (Fig 3).

The genotype-phenotype correlation was further validated at the individual parasite level using a small subset of samples (414). By subjecting these samples to bioassays followed by genotyping, we found that parasites with the SS genotype died at both 0.4 and 2 ppb azamethiphos, whereas parasites with the RR genotype survived. Parasites with the RS genotype were partly affected, as 12% died at 0.4 ppb and 33% at 2 ppb (Fig 4). Consequently, parasites of the RS genotype are still partly susceptible to azamethiphos in a concentration-dependent manner. This again demonstrates the significant role of the *Phe362Tyr* in the survival of salmon louse under azamethiphos treatment.

The data from the present study demonstrates extensively that *Phe362Tyr* is widespread throughout the Norwegian coastline. This could have happened either by the single origin of *Phe362Tyr* mutation that spread across the coast, or the mutation had multiple origins at different locations independently. The former possibility is more plausible since this mutation is the only mutation found in the *ace1a* gene in *L*. *salmonis* [7, 8]. In the literature, a number of point mutations conferring resistance to OPs have been described in arthropods [16], however none of these were found in *L*. *salmonis*. In addition, the results from our previous study [8], where we identified the *Phe362Tyr* from samples collected in 1998, indicated that this mutation might have occurred during the early 1990s when the first cases of reduced efficacy of organophosphate treatments were noted in Scotland and Norway [5]. Hence, the resistant samples would, most probably, have migrated to the nearby farms and would have propagated further.

Studies where a single point mutation could confer resistance towards a chemical have been reported in other arthropods. For example, a single mutation (*Gly119Ser*) in *ace1* has been shown to be responsible for insensitivity of the acetylcholinesterase enzyme towards the carbamate propoxur among mosquitoes that act as vectors for malaria (*Anopheles gambiae*) and West Nile virus (*Culex pipiens*) [17]. Similarly, a single mutation (*Arg81Thr*) in the beta subunit of nicotinic acetylcholine receptor (nAChR) has been reported to be associated with resistance in *Myzus persicae* towards neonicotinoids [18].

The far north (Finnmark) and far south areas (Vest Agder) of Norway are exceptions to the resistance towards azamethiphos. The significantly lower frequencies of resistance alleles in these counties attributes to limited usage of chemotherapeutants in these areas. However, the detection of a low frequency of RS genotypes in samples from these two counties indicates that even in these areas significant resistance problems would emerge rapidly if OP treatment is used.

Thus, Norway, the world’s biggest exporter of Atlantic salmon, is struggling to combat the resistant salmon louse [19]. Newer medicinal and non-medicinal strategies are being proposed and trialled in order to defeat the tough enemy. Unfortunately, the parasite seems to be on the winning end so far. The data from the present study confirms the gravity of the situation in Norway.

## Conclusion

This is the first epidemiological study on the distribution of *Phe362Tyr* across the Norwegian coast. A large cohort (n = 6658) of salmon louse from 56 different fish farms was screened for *Phe362Tyr* using TaqMan assay. This simple, rapid and reliable assay could easily detect and map the geographical distribution of azamethiphos resistance across Norway. Based on the results, we could assertively conclude that *Phe362Tyr* is a major genetic factor responsible for azamethiphos resistance in Norway with a likely single origin. This study provides a strong platform to identify resistance in its budding stage, which eventually would allow better management strategies to be designed and implemented.

## Supporting Information

S1 Table Raw data about the county sites, genotyping and bioassays of the samples enrolled. (XLSX)
